# Prevalence and factors associated with malaria, typhoid, and co-infection among febrile children aged six months to twelve years at kampala international university teaching hospital in western Uganda

**DOI:** 10.1016/j.heliyon.2023.e19588

**Published:** 2023-08-29

**Authors:** Joanitor Nakisuyi, Melvis Bernis, Andrew Ndamira, Vicent Kayini, Richard Mulumba, Pius Theophilus, Ezera Agwu, Herman Lule

**Affiliations:** a Department of Paediatrics and Child Health, Faculty of Clinical Medicine and Dentistry, Kampala International University-Western Campus, P.o. Box 71, Bushenyi, Uganda; b Department of Obstetrics and Gynecology, Faculty of Clinical Medicine and Dentistry, Kampala International University-Western Campus, P.o. Box 71, Bushenyi, Uganda; c Medical Laboratory Science Department, Kampala International University Western Campus, Bushenyi, Uganda; d Departments of Medical Microbiology and Clinical Immunology, Faculty of Medicine, Kabale University, Uganda; e Faculty of Medicine, Department of Clinical Neurosciences, Turku University Hospital and University of Turku, FI-20014, Turku, Finland

**Keywords:** Malaria, Typhoid, Co-infection, Febrile, Children, Uganda, Africa

## Abstract

**Background:**

Paediatric febrile illnesses pose diagnostic challenges in low-income countries. Western Uganda is endemic for both malaria and typhoid but the true prevalence of each individual disease, their co-infections and associated factors are poorly quantified.

**Objective:**

To determine the prevalence of malaria, typhoid, their co-infection, and associated factors amongst febrile children attending the paediatrics and child health department of Kampala International University Teaching Hospital (KIU-TH) in Western Uganda.

**Methods:**

Cross-sectional study used a survey questionnaire covering demographics, clinical and behavioural variables. We obtained blood for peripheral films for malaria and cultures for typhoid respectively; from 108 consecutively consented participants. Ethical approval was obtained from KIU-TH research and ethics committee (No. UG-REC-023/201,834). Multivariate regression analysis was performed using Stata 14.0 (StataCorp. 2015) at 95% confidence interval, regarding p < 0.05 as statistically significant.

**Results:**

Majority of participants were males 62% (n = 67), cared for by their mothers 86.1% (n = 93). The prevalence of malaria was 25% (n = 27). The prevalence of typhoid was 3.7% (n = 4), whereas the prevalence of malaria-typhoid co-infection was 2.8% (n = 3). Using treated water from protected public taps was associated with low malaria-typhoid co-infection [p = 0.04; aOR = 0.05, 95%CI [0.003–0.87], whereas drinking unboiled water from open wells increased the risk for the co-infection [p = 0.037, cOR = 17, 95%CI (1.19–243.25)].

**Conclusions:**

The prevalence of blood culture confirmed malaria-typhoid co-infection in children was lower than previously reported in serological studies. These findings emphasize the need to use gold standard diagnostic investigations in epidemiological studies. Educational campaigns should focus on the use of safe water, hygienic hand washing, and proper waste disposal; and should target mothers who mainly take care of these children.

## 1 Introduction

Febrile illnesses are still a global health challenge in the developing countries [1]. Malaria and typhoid fever are a major cause of febrile illness, responsible for 619,000 and 216,000 global deaths annually, respectively [2]. These deaths tend to double when there is dual infection [3]. Children below 15 years in sub-Saharan Africa are at risk of these two infections due to possibility of common source spread from school settings [4].

Current reports show increasing global trends of malaria during 2022 [2], and the disease burden is highest amongst low-income countries in the tropics which have seasonal variations and contaminated ground water sources [1]. The burden of malaria can be compounded with typhoid-salmonella co-infection at the interface of dry and wet seasons, linking the two disease entities [5]. Besides, the social circumstances of both diseases can be driven by malnutrition, HIV, poverty and poor sanitation [6] which are of public health concern in Uganda.

Uganda is endemic for both malaria and typhoid [7]. Therefore, clinicians in resource-constrained settings should anticipate this co-infection in children due to their overlapping clinical features [8], which makes it difficult to diagnose them accurately [9]. Diagnostic challenges prompt clinicians to treat the co-infection without laboratory confirmation, risking drug resistance [1,10]. On the one hand, failure to prescribe the relevant medications, timely poses a risk of diagnosing typhoid fever only after complications such as bowel perforation have occurred [7]. This has both legal implication and impacts on treatment outcome.

To-date, there are no lifelong protective vaccines for both malaria and typhoid due to high mutation rates [11] and inability to stimulate an immature immune system in children [12]. These challenges counteract the global target of reducing morbidity and mortality due to malaria by 90%, between 2016 and 2030 [2]. The world health organization (WHO) recommends 4 doses of “RTS,S″ malaria vaccine as part of prevention tools in children from 5 months of age, following studies that demonstrated a 30% reduction in malaria-related mortality after vaccine use in *P. falciparum* highly endemic African region [13], however, the protective effect of the vaccine wears off after 3 years [14].

According to WHO, the criterion standard for diagnosis of malaria is a blood slide whereas typhoid fever requires culture isolation of the organism, which is widely considered 100% specific [15]. Culture of the bone marrow aspirate is the most sensitive at 90% for typhoid salmonella [16], but extremely painful, which may outweigh the benefits in paediatric population. It has been shown that multiple blood cultures (>3), yield sensitivities of 73–97%, particularly larger volume (10–30 ml) [15]. Despite though, it is not routine in Uganda to obtain the mandatory three blood samples in the paediatric population and such results are often not timely available to guide prescriptions due to a backlog of samples amidst scarce human and infrastructural resources [17].

Thus due to lack of standard diagnostic tools [10], any fever in children in Uganda is primarily treated as malaria [18,19]; only to think of other causes when there is no improvement on anti-malarial drugs [7,20]. What is often available to diagnose malaria and typhoid infections in Ugandan context are rapid kits that have concerns of reduced specificity [15]. Besides, late presentation of children with fever and possible exposure to an anti-malarial or antibiotic prior to hospital visit, could result in missing such late infections even on blood smears and cultures [19,21]. This has posed threat for irrational drug prescriptions and antibiotic resistance in our tertiary hospital settings [19].

Although there are existing nation-wide interventions and published data to aid curbing malaria in Uganda [22], malaria-typhoid co-infection as a single disease entity is being overlooked in the paediatric population. Knowledge of the extent of this burden and factors associated with this co-infection are key to high index of suspicion, primary prevention, early detection, and proper integrated case management. The main objectives of the present study therefore were to determine the prevalence of: (i) malaria; (ii) typhoid; (iii) malaria-typhoid co-infection and (iv) associated factors; amongst febrile children attending the paediatrics department of Kampala International University Teaching Hospital (KIU-TH) in Western Uganda.

## 2 Methods

### 2.1 Study design

This was a cross-sectional descriptive and analytical study conducted between March–November 2019.

### 2.2 Study participants and settings

The study involved children aged between 6 months to twelve years who presented with fever at the department of paediatrics and childcare of Kampala International University Teaching Hospital (KIU-TH). All eligible children with fever at the paediatric department of KIU-TH including outpatients, in-patients, and emergency wards; were consecutively recruited until the desired sample size was realised. This was intended to generate a sample size large enough to relate the findings to the population.

The study site is the main teaching hospital for Kampala International University Schools of Medicine and Allied Health, located in Ishaka Municipality, Bushenyi District of Western Uganda. The hospital is a 700-bed capacity, providing emergency, out and in-patient specialised paediatrics and child health care. According to the Uganda Bureau of Statistics [23], the hospital provides diagnostic and therapeutic services to over 16,646 catchment population. This malaria endemic region has two rainy seasons, beginning March to May, and September to November, during which malaria and diarrhoeal infections peak.

According to Uganda Bureau of Statistics [23], the population of children between six months to 12 years in Bushenyi district is about 45.9%; of which 7.2% do not attend school; 88.1% attend primary school, while the illiteracy rate is reported to be 12.1%. Reportedly, over 95.8% of the district's population own at least one mosquito net; only 16.1% have access to piped water whereas 6.8% use bore holes. In addition, up to 0.6% of the districts' population do not have access to any toilet facility and practice open defecation while only 23.1% practice proper solid waste disposal and 95.7% are not living in descent dwellings. Findings from a study on spatio-temporal distribution of typhoid showed that the highest disease burden was recorded in central, followed by western and south-western Uganda, and least in eastern and northern parts [24].

### 2.3 Sample size calculation

Being across sectional study where the proportion (P) was the parameter of interest, and using non random sampling, the sample size was calculated using modified Daniel's formula [25].

**Objective 1:** The prevalence of malaria in children in Bushenyi District in Western Uganda had been reported to be 3.5% [26] and therefore P = 0.035. Assuming a statistical power of 80% at 95% CI, the resulting sample was 106.

**Objective 2:** Based on the study done at KIU-TH in Western Uganda, the prevalence of typhoid fever in children was reported to be 2.76% [27]. Substituting 0.0276 for P and assuming a statistical power of 80% at 95% CI, the resulting sample was eighty-four.

**Objective 3:** Based on the Tanzania study the prevalence of malaria-typhoid co-infection was reported to be 3.5% [20]. Substituting 0.035 for P, and assuming a statistical power of 80% at 95% CI, the resulting sample was 106.

Therefore, a minimum sample size of 106 was considered adequate to address all the study objectives. Detailed sample size calculations are available as supplementary file 1.

### 2.4 Inclusion criteria

All children aged between 6 months and 12 years with fever were recruited into the study.

### 2.5 Exclusion criteria

Children whose parents or legally authorised representatives declined consent during study period were excluded. Patients with a history of antibiotic and/or anti-malarial treatment within 2 weeks prior to admission, and those on malaria prophylaxis or long-term antibiotics were excluded from the study to minimise false negative results.

### 2.6 Study procedure

Malaria cases were stratified as uncomplicated or severe based on clinical symptoms and number of malaria parasites as observed under a microscope [28]. This stratification was for the purposes of proper case management by the attending clinicians. Blood samples for typhoid salmonella culture were collected from eligible participants with a positive blood slide for malaria.

**Fig. 1:**
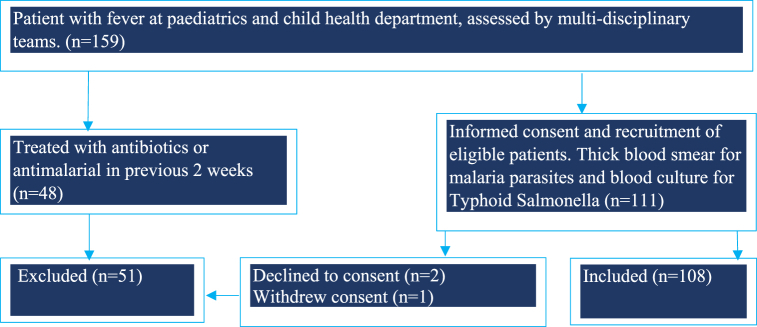
Showing flow of participants.

Recruitment of study participants was conducted at the paediatrics and child health (emergency, outpatient, and inpatient) units of KIU-TH, after emergency resuscitation (if deemed necessary by the attending clinician). Every respondent or legally authorised representative was explained to the purpose of the study to endorse an informed consent document with a signature or thumb print. A pretested coded check list of parameters of interest specially designed for this purpose was then administered by the investigators. A complete history of associated symptoms such as nausea, loss of appetite, headache, abdominal and join pain, physical examination and relevant laboratory investigations was conducted and findings of interest were recorded on the data tool. In general, patients at paediatric department are received and triaged by the medical team on duty. The first contact clinician is a general doctor who then consults a paediatric resident, paediatrician, or infectious disease specialist when there is need. The team routinely carries out several ward rounds in a day to review laboratory results and determine if there is need to amend the initial treatment decisions**.** The recruitment process and flow of participants is summarised in (Fig. 1).

### 2.7 Laboratory procedures

All laboratory analyses were conducted at the microbiology laboratory of KIU-TH. Patients were sent at the laboratory reception where they were assigned a unique laboratory number after registration, followed by blood sample collection. Caretakers to participants or legally authorised representatives were asked to give written informed consent for both specimen collection and subsequently to answer a brief questionnaire in their local language for the illiterate.

### 2.8 Collection of samples for malaria blood slide

The ring finger was cleaned using an anti-septic solution (chlorhexidine) and allowed to dry, and then pricked with a sterile lancet. The first drop of blood was cleaned with a dried cotton wool and finger was squeezed to allow a drop of blood to flow on the centre of a clean, dry, grease free glass slide. A clean glass rod was used to spread the blood in a circular motion to make a thick blood film such that the back of the watch can be read. The prepared thick blood film was allowed to air dry in accordance with [29].

### 2.9 Collection of blood sample for culture of salmonella

The skin at a chosen site for venipuncture was cleaned using an antiseptic solution. The area was allowed to dry prior to venipuncture. A non-touch technique was used to draw three mls of venous blood that was transferred into brain heart infusion broth after disinfection of the rubber septum using an antiseptic solution. The culture bottle was labelled with the participant code number and then taken to the laboratory immediately. Following arrival at the laboratory, each specimen was registered in the appropriate record book and incubated at 37ᵒC for 7days in accordance with [30]. Samples collected in the night also underwent a similar process since the laboratory is easily accessible and within the hospital. The specimen was prepared as follows.

### 2.10 Thick blood smears staining

The dried thick blood smears were prepared in accordance with a method described by Ref. [31]. The “plus subsystem” was used to quantify the malaria parasites in accordance with centre for disease control criteria [32]. This was intended to guide clinical management.

### 2.11 Blood culture and gram staining for morphology

After 7 days of incubation, blood samples with growth were sub-cultured on Salmonella-Shigella agar (SSA) under class II biosafety cabinet and incubated at 37ᵒC for 18–24 h. Cultures were re-incubated after first 24 h without growth for up to 72 h before reporting no growth. Cultures with growth were observed for colony characteristics. Colonies were picked with the help of sterile wire loop and smears were made by emulsifying the colony with a drop of normal saline on a clean dried slide. Gram-staining was done to observe the morphology features under a microscope and *Salmonella* colonies were identified in accordance with De et al. [33].

### 2.12 Quality control

All slides and gram stains were interpreted by two independent laboratory technologist who were blinded of the patient's history. In case of disagreement, a professor of medical microbiology and parasitology (EA) was consulted, and his decision was considered final. Each of the slides were compared with a standard positive malaria blood slide already available in the hospital laboratory. Each suspected *Salmonella* isolate from research participant was compared with a standard *Salmonella* organism. All the positive samples of isolated Salmonella were taken for external quality control as blind duplicate samples at the nearby Mbarara Regional Referral Hospital for validation.

### 2.13 Data collection methods and study variables

We collected data using investigator administered pre-tested questionnaire designed in English and local language (Runyankole). We obtained data on independent variables including fever, abdominal pain, vomiting, and loss of consciousness. The data tool also captured information on social circumstances which based on previous literature [34,35]; were presumed to have an influence on disease transmission including: socio-demographic factors (age, maternal level of education, school going status of the child); behavioural factors (source of drinking water, hand washing practices, definitive human waste disposal); and awareness of preventive measures for the two infections. This method had been validated to be effective in similar study settings [28].

### 2.14 Validity and reliability of data collection instrument

The pre-test study was conducted at Lugazi Health Centre IV. We used content validity index in which five participants who were not part of the sample population, were given the questionnaire. A measure of inter-participant agreement was determined. A Cronbach's co-efficient alpha of more than 0.8 was considered to imply that the items on the questionnaire were reproducible and consistent.

### 2.15 Data analysis

Data was entered into Microsoft Excel (version 2010) and exported to Stata software version 14.1 (StataCorp. 2015. Stata Statistical Software: Release 14. College Station, TX: StataCorp LP) for cleaning and analysis. The participants’ socio-demographic, behavioural and clinical characteristics are summarised using frequencies and percentages in tables. The mean and standard deviation were used for continuous variables that were normally distributed otherwise the median and inter-quartile range were used. We used the modified Poisson regression (with robust standard errors) model to determine factors associated with malaria-typhoid co-infection. Factors with medical plausibility and those with p < 0.2 at bivariate analysis were considered for multivariate analysis. At multi-variate analysis, confounding and effect modification (interaction) were assessed at cut-off of 15%. The factors with p < 0.05, in the final model were statistically significant. The measures of association are reported as odds ratios (OR), with corresponding 95% CI and p-values.

## 3 Results

By the end of the study period, a total of 159 participants were seen at the peadiatric and child health department and 108 were eligible for inclusion and analyses (Fig. 1). All patients with uncomplicated malaria were treated with artemether-lumefantrine combination therapy (ACT) for 3 days whereas those with complicated malaria were treated with intravenous artesunate 3 mg/kg (weight <20 kg) and 2.4 mg/kg (weight >20 kg) at 0, 12, 24 h then every 24 h until they could tolerate oral ACT in accordance with WHO guidelines [36]. Further, patients confirmed to have typhoid received intravenous ceftriaxone 50 mg/kg/day (maximum 2 g) for 7 days in accordance with a local hospital protocol.

### 3.1 Socio-demographic characteristics of febrile children attending paediatric department at KIU-TH

Of the 108 participants, majority were below the age of 1 year 38.9% (n = 42). Over 85.2% (n = 92) of the legal guardians were married and it is largely mothers who took care of these children 86.1% (n = 93). Male children dominated their female counterparts (62.0%; n = 67) vs. 38.0%; n = 41). Majority 73.2% (n = 79) were school going, although their mothers were illiterate (40.7%; n = 44); working as peasant farmers 43.5% (n = 47) or stay-at-home spouses 20.4% (n = 22). Majority 55.6% (n = 60) lived in semi-permanent houses as shown in (Table 1).

**Table 1:** Socio-demographic characteristics of febrile children attending paediatric department at KIU-TH.

Variable	Frequency (n)	Percentage (%)
**Sex**		
Male	67	62.0
Female	41	38.0
**Age category**		
<1year	42	38.9
1–3	24	22.2
4–6	18	16.7
7–9	14	13.0
10–12	10	9.3
**School going status**		
Yes	79	73.2
No	29	26.9
**Education level of mother**		
None	44	40.7
Primary	12	11.1
Secondary	32	29.6
Tertiary	20	18.5
**Education level of father**		
None	19	17.6
Primary	5	4.6
Secondary	54	50.0
Tertiary	30	27.8
**Caretaker of child**		
Father	1	0.9
Mother	93	86.1
Sibling	3	2.8
Maid	6	5.6
Others	5	4.6
**Type of house**		
Permanent	48	44.4
Semi-permanent	60	55.6

### 3.2 Behavioural characteristics of febrile children attending peadiatric department at KIU-TH

Over 61.0% (n = 66) of the children and or their guardians seldom washed their hands before eating food whereas over 48.0% (n = 52) of them frequently wash their hands without soap. Additionally, only 48.2% (n = 52) of the children could wash their hand after use of toilet/latrine. Hygienically, about 2.0% practiced open defecation, while 82.4% (n = 89) of the participants used latrine/toilets (Table 2).

**Table 2:** Behavioural characteristics of febrile children attending paediatric department at KIU-TH.

Variable	Frequency (n)	Percentage (%)
**Washing hands before feeding**		
No	1	0.9
Yes	41	38.0
Sometimes	66	61.1
**What is used when washing hands**		
None	4	3.7
Plain water only	15	13.9
Water with soap	37	34.3
Sometimes with plain water/without soap	52	48.2
**Human waste disposal**		
Open defecation	2	1.9
Latrine/toilet	89	82.4
Both open defecation and latrine/toilet	17	15.7
**Washing hands after defecation**		
No	18	16.7
Yes	52	48.2
Sometimes	38	35.2

Most participants, 30.6% (n = 33) had their source of water from public taps followed by boreholes 26.9% (n = 23). In our study population, 3.7% (n = 4) used unboiled water. Approximately 83.3% (n = 90) of the participants had heard of malaria prevention whereas 71.3% (n = 77) had heard about typhoid prevention programs either on radio, television, or community (Table 3).

**Table 3:** Source of water and media awareness about malaria and typhoid amongst febrile children attending paediatric department at KIU-TH.

Variable	Frequency (n)	Percentage %)
**Source of water**		
Open well	11	10.2
Public borehole	29	26.9
Public tap	33	30.6
Family Tap	1	0.9
Borehole and public tap	23	21.3
Open well and borehole	4	3.7
Open well and shared public tap	4	3.7
Others	3	2.8
**Status of drinking water**		
Boiled	94	87.0
Unboiled	4	3.7
Both boiled and unboiled	10	9.3
Stagnant water around the house		
No	77	71.3
Yes	31	28.7
**Ever heard about a programme on malaria prevention**		
No	18	16.7
Yes	90	83.3
No		
**Ever heard about a programme on Typhoid prevention**		
No	31	28.7
Yes	77	71.3

### 3.3 Prevalence of malaria, typhoid and malaria-typhoid co-infections among febrile children attending paediatric department at KIU-TH

The prevalence of malaria and typhoid were 25.0% (27/108) and 3.7% (n = 4) respectively whereas the co-infection was prevalent in 2.8% (n = 3) (Table 4).

**Table 4:** Prevalence of malaria, typhoid and their co-infection among febrile children attending paediatric department at KIU-TH.

Variable	Result	Frequency n (%)	95%CI
Malaria	Negative	81(75.0)	65.8–82.4
	Positive	27(25.0)	17.6–34.2
Typhoid	Negative	104(96.3)	90.4–98.6
	Positive	4(3.7)	1.4–9.6
Malaria-typhoid co-infection	Negative	105(97.2)	91.6–99.1
	Positive	3(2.8)	0.9–8.4

The most affected age groups for malaria and malaria-typhoid co-infection were (7–9) and (10–12) years respectively (Table 5).

**Table 5:** Age specific prevalence of malaria, typhoid and malaria-typhoid co-infection among febrile children attending paediatric department at KIU-TH.

Variable	Age group in yrs. (n)	Frequency (n)	Percentage (%)	95% CI	P-value
Malaria					
	<1(n = 42)	9	21.4	11.2–37.0	1.00
	1-3(n = 24)	6	25.0	11.0–47.4	0.74
	4-6(n = 18)	4	22.2	7.7–49.4	0.95
	7-9(n = 14)	5	35.7	13.7–66.0	0.29
	10-12(n = 10)	3	30.0	7.6–69.0	0.57
Typhoid					
	<1(n = 42)	0	–	–	–
	1-3(n = 24)	0	–	–	–
	4-6(n = 18)	0	–	–	–
	7-9(n = 14)	1	7.1	0.7–44.0	0.17
	10-12(n = 10)	3	30.0	8.0–69.0	–
Malaria-Typhoid co-infection					
	<1(n = 42)	0	–	–	–
	1-3(n = 24)	0	–	–	–
	4-6(n = 18)	0	–	–	–
	7-9(n = 14)	1	7.1	0.7–44.0	0.37
	10-12(n = 10)	3	20.0	4.0–62.0	–

### 3.4 Clinical characteristics of febrile children with malaria and typhoid attending paediatric department of KIU-TH

The mean duration of fever was 4.4 days + 3.1 (SD). The commonest symptoms were vomiting 44.4% (n = 48), abdominal pain 34.3% (n = 37), and headache 17.7% (n = 19). Based on mid-upper arm circumference (MUAC) screening, majority 93.3% (n = 95) had normal nutritional status for their age groups in accordance with WHO classification [37] and only 9.3% (n = 10) were HIV positive (Table 6).

**Table 6:** Clinical characteristics of febrile children attending paediatric department of KIU-TH.

Symptoms	Variable	Peripheral Blood Smear		Blood Culture	
		Positive n (%)	Negative n (%)	Positive n (%)	Negative n (%)
Headache	No	24(88.9)	65(80.2)	1(25.0)	88(84.6)
	Yes	3(11.1)	16(19.8)	3(75.0)	16(15.4)
Nausea and Vomiting	No	13(48.1)	47(58.0)	1(25.0)	69(66.3)
	Yes	14(51.9)	34(42.0)	3(75.0)	35(33.7)
Abdominal pain	No	17(63.0)	54(66.7)	0(0.0)	71(68.3)
	Yes	10(37.0)	27(33.3)	4(100.0)	33(31.7)
Joint Pain	No	23(85.2)	75(92.6)	2(50.0)	96(92.3)
	Yes	4(14.8)	6(7.4)	2(50.0)	8(7.7)
Signs	None	8(29.6)	22(27.2)	0(0.0)	30(28.8)
	Dehydration	5(18.5)	22(27.2)	1(25.0)	26(25.0)
	Seizures	0(0.0)	4(4.9)	0(0.0)	4(3.8)
	Tachycardia	0(0.0)	6(7.4)	0(0.0)	6(5.8)
	Tachypnoea	0(0.0)	2(2.5)	0(0.0)	2(1.9)
	Splenomegaly	1(3.7)	2(2.5)	0(0.0)	3(2.9)
	Others	13(48.1)	23(28.4)	3(75.0)	33(31.7)
Nutrition status	Normal	23(85.2)	72(88.9)	3(75.0)	92(88.5)
	Moderate malnutrition	3(11.1)	7(8.6)	1(25.0)	9(8.7)
	Severe malnutrition	1(3.7)	2(2.5)	0(0.0)	3(2.9)
HIV Status	Positive	1(3.7)	9(11.1)	0(0.0)	10(9.6)
	Negative	26(96.3)	72(88.9)	4(100.0)	94(90.4)

### 3.5 Factors associated with malaria-typhoid co-infection among febrile children attending paediatric department at KIU-TH

Before adjustment, children who were being taken care of by their mothers were 96.0% less likely to have malaria-typhoid co-infection compared to those being taken care of by their fathers [p = 0.028; cOR = 0.04, 95% CI (0.003–0.71)], however this association did not remain valid upon adjustment for confounding [p = 0.33; aOR = 0.14, 95%CI (0.003–7.33)].

Children who reported taking unboiled drinking water from open wells were 17 times more likely to get malaria-typhoid co-infection [p = 0.037, cOR = 17, 95%CI (1.19–243.25)]. Children whose source of water was public taps were 97.0% less likely to have malaria-typhoid co-infection compared to those who used open wells [p = 0.015, cOR = 0.03, 95%CI [0.02–0.51]. This association remained statistically significant even after adjustment for confounding [p = 0.04; aOR = 0.05, 95%CI (0.003–0.87)]. There was no statistically significant association between malaria-typhoid co-infection and gender, level of education, type of accommodation and school going status (Table 7).

**Table 7:** Bivariate and multivariate analysis of factors associated with malaria-typhoid co-infection among febrile children attending paediatric department at KIU-TH.

Variable	cOR	95%CI	p-value	aOR	95%CI	p-value
**Sex**						
Male	1.00	–	–			
Female	3.38	0.30–38.56	0.33			
**School going status**						
No	1.00	–	–			
Yes	1.38	0.12–15.76	0.80			
**Parent education level**						
None	1.00	–	–			
Primary	1.73	0.10–30.45	0.71			
Secondary	0.61	0.04–10.39	0.74			
Tertiary	–	–	–			
**Caretaker**						
Father	1.00	–	–	1.00	–	–
Mother	0.04	0.00–0.71	0.03	0.14	0.00–7.33	0.33
**Type of house**						
Semi-permanent	1.00	–	–			
Permanent	0.39	0.00–4.43	0.45			
**Source of drinking water**						
Open well	1.00	–	–	1.00	–	–
Public taps	0.03	0.02–0.51	0.02	0.05	0.00–0.87	0.04
**Status of drinking water**						
Boiled	1.00		–			
Unboiled	17.00	1.19–243.25	0.04			
**Own mosquito net**						
NO	1.00	–	–			
YES	0.21	0.12–2.53	0.22			
**Uses a mosquito net**						
NO	1.00	–				
YES	0.03	0.00–0.43	0.01			
**Holes in mosquito net**						
NO	1.00	–				
YES	1.21	0.11–13.94	0.88			
**Treated mosquito net**						
YES	1.00	–	–			
NO	2.11	0.18–25.14	0.55			
**Stagnant water around home?**						
NO	1.00	–				
YES	5.24	0.46–60.03	0.18			
**Prevention for malaria**						
Insecticides	1.00	–	–			
Mosquito nets only	0.09	0.01–1.67	0.11			
Both insecticides and mosquito nets	0.27	0.02–5.03	0.33			
**What time are windows closed?**						
4–6pm	1.00	–				
6–7pm	–	–				
7–8pm	3.11	0.19–52.08	0.43			
>8pm	9.83	0.54–178.00	0.12			
**Ever heard of typhoid program?**						
NO	1.00	–	–			
YES	0.08	0.07–9.16	0.86			

## 4 Discussion

### 4.1 Prevalence of malaria amongst febrile children attending paediatric department of KIU-TH

The first objective of the study was to determine the prevalence of malaria amongst febrile children attending the paediatric department of KIU-TH which was found to be 25.0%. This prevalence is lower than 36.5% reported in an Ethiopian study [35], though higher than the 3.5% [38] and 12.0% [22] previously reported in Western and South Western Uganda respectively. The current figure is also higher than the Ugandan National average of 19.0% [39]. This discrepancy could be arising from differences in inclusion criteria, our study having recruited only those who were febrile, including those above 5 years. There has been also concerns that regular reports from Uganda Health Management and Information System (HMIS) suffer inaccuracies; including underreporting of fevers, since only episodes covered by the national public health system are captured, amidst lack of laboratory confirmation [22]. In addition, our relatively higher prevalence could also be related to seasonality, having conducted the study in two rainy and one dry season as opposed to the national average that is based on a single calendar year [39].

However our findings show a significant reduction in malaria prevalence from a previously reported Ugandan National average of 42.0% in 2009 [40]. This reduction could depict successful National malaria control programmes such as indoor residual spray and distribution of free insecticide treated mosquito nets for vector control. The most affected age group in our study population was 7–9 years. In a similar study in Northern Uganda, this age group was the most affected at 61.8% [41]. This could be due to increased outdoor activity in this age group, but whether mostly the exposure is at school or home deserves further investigation.

### 4.2 Prevalence of typhoid amongst febrile children attending paediatric department of KIU-TH

The second objective of the study was to determine the prevalence of typhoid amongst febrile children attending the paediatric department of KIU-TH, based on blood cultures. The study established the prevalence at 3.7% and all affected participants were aged 7–12 years. This blood culture based prevalence is comparable to 2.8% [27] previously reported at KIU-TH and to 2.3% reported at Mulago National Referral Hospital [42]. Also our low prevalence compares well to 0.5%–5.0% reported by Birhanie et al. [35] and Habte et al. [43] respectively in Ethiopia.

Previously in Uganda, typhoid infection based on blood cultures has been largely studied only during outbreaks rather than routine in the paediatric population. Ranges between 2.6% and 22.6% were reported among adults during Kasese outbreak in Western Uganda [44]. In a retrospective study amongst all febrile patients attending clinics in Bushenyi district, the overall prevalence was reported to be 36.6%, affecting mainly 10–29 year olds of low income class [45], however this was a Widal Agglutination serological based study with sensitivity and specificity concerns, amidst data quality constraints of retrospective studies. Serological tests as opposed to blood cultures have been found to give higher prevalence rates of typhoid fever, resulting from false positive results in Nigeria [46], India [47] and Pakistan [48]. In an Ethiopian study, typhoid fever was prevalent in 19.0% based on serological Widal test as opposed to 0.5% based on blood culture [35], emphasising the need for extending laboratories with capacity to do blood cultures for proper diagnosis. In conformity with our study findings, a blood culture study in Cameroon found typhoid fever prevalence of 2.5% amongst febrile patients. Other studies in low and middle income countries have found typhoid prevalence lowest amongst children below 4 years and above 15 years [46,49] and highest amongst school going age group of 5–10 years [48]. The higher Typhoid burden in the later age group could be due to common source infections from public boreholes in our primary schools setting, alongside poor hand hygienic practices.

### 4.3 Prevalence of malaria-typhoid co-infection amongst febrile children attending paediatric department at KIU-TH

The third objective of the study was to determine the prevalence of malaria-typhoid co-infection amongst febrile children attending the paediatric department of KIU-TH. The prevalence of malaria-typhoid co-infection based on microscopy and blood culture respectively in our study population was found to be at 2.8%. Our co-infection rates are comparable to 3.5% reported in a Tanzanian study among children below 15 years [20] and to 2.5% in an Indian study [47]. Contrary to the findings of Birhanie et al. [35], all cases in the present study were between 7 and 12 years as opposed to 2–5 years. However, the prevalence of this co-infection in our study is lower than 6.5% reported in an Ethiopian study of blood cultures [35], although the later involved a general population; including participants above 12 years.

The co-infection rates in our study are far lower that what has been reported previously in serological studies. In their serological study in Western Uganda, Agwu et al. [50] reported a co-infection rate of 20.9% which was comparable to 18.3% in a Nigerian study that used serological tests in a general population [46]. The inclusion of general population should control for confounding from comorbidities such as HIV/AIDs that have been shown to be associated with higher rates of both malaria and typhoid [50], otherwise the resulting high prevalence of malaria-typhoid co-infection could be overestimated. In a similar study in Sierra Leon, there was no association between having malaria and typhoid fever, but presence of fever was more associated with *Salmonella Typhi* compared to *Plasmodium* parasites [51]. A consensus on age specific-blood culture-based reporting of this co-infection amongst researchers, could thus address variability of findings in the future studies. Research on malaria-typhoid co-existence is critical due to a compelling body of evidence to suggest that inherent immunological responses such as hemolysis caused by acute malaria infection is an independent risk for superimposed typhoid infection which would otherwise be “silent” to a level detectable by blood culture [34,35,52].

### 4.4 Factors associated with malaria-typhoid co-infection amongst febrile children attending paediatric department at KIU-TH

The fourth objective of the study intended to determine the factors associated with Malaria-Typhoid co-infections amongst febrile children. Upon adjusting for confounders, we found that the most crucial factor influencing this co-infection was the source of water. Using treated water from protected public taps was associated with low malaria-typhoid co-infection (p = 0.04) whereas drinking unboiled water from open public wells increased the risk for the co-infection (p = 0.037). Capturing such history could be made a routine element of screening children presenting with febrile illnesses in our settings.

Other Ugandan authors have pinned contaminated water and food as main driving factors for typhoid infection [44], although their main focus had been on adult population. In a similar Ethiopian study, using non treated water from open sources such as springs and wells was associated with blood culture confirmed typhoid fever, especially amongst rural dwellers [43]. Contrary to findings of Khan et al. [48] in Pakistan, we found no significant association between the child's school going and malaria-typhoid co-infection in the present study despite the fact that all cases were within 7–12 years; a typical school going age group. Other studies attribute malaria-typhoid co-infection in school going age to increased outdoor activity as well as poor hand washing habits in absence of parental supervision [35]. In our study, over 61.0% of the children seldom washed their hands before handling food, whereas 48.0% of them did so but without soap. These statistics coupled with open defecation and failure to wash hands after visiting toilets demonstrated in the present study, warranty urgent behavioural change campaigns.

### 4.5 Study strengths and limitations

This study boosts of several strengths. First, the investigations used in the present study i.e., blood culture and blood side microscopy are considered gold standard in definitive diagnosis of typhoid and malaria respectively. Secondly, being a cross-sectional study, the level and quality of completeness of data could easily be controlled. Moreover, the data tool used was not only specifically designed for this study but also validated for reliability. Lastly, all positive cases of Salmonella were externally cross-examined and confirmed by an external reference laboratory.

However, there were some limitations in this study. First, although blood slide and blood culture are considered gold standard for diagnosing malaria and typhoid respectively, in exceptional cases, malaria parasites may not be captured in peripheral blood smears even in presence of severe infection due to sequestration of parasitized cells in deep capillary beds. Secondly, the reported isolated case of typhoid fever in the present study was included based on presence malaria pigment in circulating neutrophils and monocytes despite having no malaria parasites in setting of suspected malarial infection. In addition, the sensitivity of blood cultures for typhoid salmonella is intrinsically moderate at only 85.0–90.0% [16,53]. Lastly, the prevalence which was used in our sample size calculation was just an estimate for the proportionate split based on a single previous study, assuming normal approximation. As such, a maximum variation of assuming 0.5 proportion could have yielded a more “conservative” sample size, higher statistical power, and more precise confidence interval estimates of the true population [54]. These factors together with our consecutive recruitment could limit the generalisability of the findings.

## 5 Conclusions and implication of results

The prevalence of malaria was high in our study population compared to the national average [39]. The prevalence of blood culture confirmed typhoid fever and malaria-typhoid co-infection were not as high as previously reported based on serological studies [50]. The co-infection was clustered mainly among children aged 7–12 years. Although the prevalence of co-infection was low to conclusively discern on the risk factors, using treated water from protected public taps seemed protective whereas consuming unboiled water from open public wells was a statistical risk. These findings justify the routine standardised testing for either infection amongst febrile children, to avoid irrational antibiotic prescriptions [19] and complications related to late diagnosis. Health worker and community driven educational campaigns should focus on use of safe water, hygienic hand washing practices and proper waste disposal, and should target mothers who mainly take care of these children.

## Author contribution statement

Joanitor Nakisuyi: Conceived and designed the experiments; Performed the experiments; Contributed reagents, materials, analysis tools or data; Wrote the paper. Melvis Bernis; Andrew Ndamira; Vicent Kayini: Contributed reagents, materials, analysis tools or data. Richard Mulumba: Conceived and designed the experiments. Pius Theophilus; Ezera Agwu: Performed the experiments; Contributed reagents, materials, analysis tools or data. Herman Lule: Conceived and designed the experiments; Performed the experiments; Analyzed and interpreted the data; Contributed reagents, materials, analysis tools or data; Wrote the paper.

## Data availability statement

Data will be made available on request.

## Ethics approval and consent to participate

The study strictly followed the Uganda National Courncil for Science and Technolgy Guidelines (2014) on research involving use of human participants and in accordance with the Declaration of Helsinki [56]. Ethical approval was obtained from the School of Medicine Research and Ethics Committee of Kampala International University, Western Campus (No. UG-REC-023/201,834). Informed consent was sought from all participants and or their legally authorised representatives who endorsed their signatures or thumb prints on the consent form document, having been made to understand the risks and benefits of the study. All participants were free to withdraw their consent at any stage of the study. Withdrawal of consent by any patient did not affect the quality of treatment or impinge on their entitlements. All laboratory results were immediately availed to the guardians and attending clinicians to guide treatment.

## Consent for publication

Not applicable.

## Declaration of competing interest

The authors declare that they have no known competing financial interests or personal relationships that could have appeared to influence the work reported in this paper.

## List of abbreviations

CDC: Centre for Disease Control and Prevention
GTS: Global Technical Strategy for Malaria
Hb: Haemoglobin
HIV: Human Immunodeficiency Virus
ICCM: Integrated Community Case Management
KIU-TH: Kampala International University Teaching Hospital
MIS: Malaria Indicator Survey
P·I: Patient Information
SDG: Sustainable Development Goals
SSA: Salmonella Shigella Agar
UBOS: Uganda Bureau of Statistics
WHO: World Health Organization
LMICs: Low to Middle Income Countries

## Operational definitions

**Fever:** Refers to rectal temperature equals to or above 38ᵒC [51]. However, in this study, because most guardians reported self-prescription of antipyretics for their children prior hospital visits, all children with temperatures above 37.4 ᵒC were considered febrile.

**Malaria:** An acute or chronic illness characterized by paroxysms of fever, chills, fatigue, anaemia and splenomegaly in presence of malaria parasites [2].

**Uncomplicated Malaria**: A patient presenting with symptoms of malaria and a positive blood slide for Malaria but with no features of severe malaria [2].

**Severe Malaria:** A patient with one or more of the following features occurring in absence of an identified alternative cause and in the presence of *P. falciparum*: impaired consciousness (Blantyre coma score<3 in children); prostration (generalised weakness so that the person is unable to sit, stand or walk without assistance); multiple convulsions (more than two episodes within 24 h); Acidosis (A base deficit of >8mEq/L or if not available, plasma bicarbonate level of <15 mmol/L or venous plasma lactate≥5 mmol/L, or severe acidosis manifesting clinically as respiratory distress (rapid deep laboured breathing); hypoglycaemia (blood or plasma glucose<2.2 mmol/L(<40 mg/dL), severe Malarial anaemia (Hb ≤ 5 g/dL or hematocrit of ≤15% in children <12 years of age (<7 g/dL and <20% respectively in adults with a parasite count of >10,000/μL); renal impairment (plasma or serum creatinine >265 μmol/L (3 mg/dL) or blood urea> 20 mmol/L; jaundice(plasma or serum bilirubin>50 μmol/L (3 mg/dL) with a parasitic density> 100,000/μL; pulmonary edema (radiological confirmation or oxygen saturation<92% on room air with respiratory rate> 30/min often with chest in drawing and crepitation on auscultation; significant bleeding (including recurrent or prolonged bleeding from the nose, gums or vein puncture sites, hematemesis or melena); shock (capillary refill ≥3s or temperature gradient on leg, systolic blood pressure of <70 mmHg with evidence of impaired perfusion (cold peripheries); hyper-parasitemia (>10% *P. falciparum,* > 20,000/μL *P. vivax* or >100,000/μL *P. knowlesi)* [55].

**Typhoid fever:** A patient presenting with history of persistent gradual fever, abdominal pain, bloating, red spotted rash and positive blood culture suggestive of *Salmonella typhi* [15].

**Confirmed Malaria-Typhoid co-infection:** A patient meeting criteria for malaria based on microscopy with a positive blood culture suggestive of *Salmonella typhi or paratyphi* [15].
