# Supplementary material for: Prevalence and factors associated with malaria, typhoid, and co-infection among febrile children aged six months to twelve years at kampala international university teaching hospital in western Uganda
Source: Heliyon. 2023 Aug 29;9(9):e19588. doi: 10.1016/j.heliyon.2023.e19588 (PMC10558847; doi:10.1016/j.heliyon.2023.e19588)
Supplement: Supplementary file 1 [file mmc1.docx]

**Detailed sample size calculations**

Being across sectional study where the proportion (P) was the parameter of interest, and using non random sampling, the sample size was calculated using modified Daniel’s formula^1^.

**Objective 1:** The prevalence of malaria in children in Bushenyi District in Western Uganda had been reported to be 3.5%^2^ and therefore P=0.035. Assuming a statistical power of 80% at 95% CI, the resulting sample was 106.

$n=\frac{\left( z_{\alpha}+z_{\beta} \right)^{2}p(1-p)}{e^{2}}$*;* α=0.05, e=5%; β=0.2 at 80% power;$z_{\alpha}=1.96$ $z_{\beta}=0.84$

By substitution; $n=\frac{\left( 1.96+0.84 \right)^{2}x 0.035(1-0.035)}{{0.05}^{2}}$*=* 106

**Objective 2:** Based on the study done at KIU-TH in Western Uganda, the prevalence of typhoid fever in children was reported to be 2.76%^3^. Substituting 0.0276 for P and assuming a statistical power of 80% at 95% CI, the resulting sample was eighty-four$.$

$n=\frac{\left( z_{\alpha}+z_{\beta} \right)^{2}p(1-p)}{e^{2}}$*;* α=0.05, e=5%; β=0.2 at 80% power;$z_{\alpha}=1.96$; $z_{\beta}=0.84$

By substituting; $n=\frac{\left( 1.96+0.84 \right)^{2}x 0.0276(1-0.0276)}{{0.05}^{2}}$*=* 84

**Objective 3:** Based on the Tanzania study the prevalence of malaria-typhoid co-infection was reported to be 3.5%^4^. Substituting 0.035 for P, and assuming a statistical power of 80% at 95% CI, the resulting sample was 106.

$n=\frac{\left( z_{\alpha}+z_{\beta} \right)^{2}p(1-p)}{e^{2}}$*;* α=0.05, e=5%; β=0.2 at 80% power;$z_{\alpha}=1.96$; $z_{\beta}=0.84$

By substituting; $n=\frac{\left( 1.96+0.84 \right)^{2}x 0.035(1-0.035)}{{0.05}^{2}}$*=*106

Therefore, the larger sample size of 106 was considered adequate to address all the study objectives.

**References:**

1. Charan J, Biswas T. How to calculate sample size for different study designs in medical research? *Indian J Psychol Med*. 2013;35(2):121-126. doi:10.4103/0253-7176.116232

2. Roh ME, Oyet C, Orikiriza P, et al. Asymptomatic Plasmodium infections in children in low malaria transmission setting, southwestern Uganda. *Emerg Infect Dis*. Published online 2016. doi:10.3201/eid2208.160619

3. Kiwungulo B, Pius T, Nabaasa S, ... Health Education is a Key Pillar in Reducing Prevalence of Typhoid among Febrile Patients in Peri-Urban Western Uganda: A Cross-Sectional Study. *Int …*. 2017;5(6):130-135. doi:10.17354/ijss/2017/465

4. Chipwaza B, Mhamphi GG, Ngatunga SD, et al. Prevalence of Bacterial Febrile Illnesses in Children in Kilosa District, Tanzania. *PLoS Negl Trop Dis*. 2015;9(5). doi:10.1371/journal.pntd.0003750
